# Pattern of Treatment Initiation and Outcomes for Patients With Metastatic Non-small Cell Lung Cancer in Ontario

**DOI:** 10.7759/cureus.24605

**Published:** 2022-04-29

**Authors:** Jerry W Ding, Abdulkadir A Hussein, Zhong Ren Huang, Kamran Ehsan, Devinder Moudgil, Swati Kulkarni

**Affiliations:** 1 Medical Oncology, Windsor Regional Hospital Cancer Program, Windsor, CAN; 2 Medicine, Schulich School of Medicine & Dentistry, Windsor, CAN; 3 Mathematics & Statistics, University of Windsor, Windsor, CAN; 4 Oncology, Windsor Regional Hospital Cancer Program, Windsor, CAN

**Keywords:** overall survival rate factors, distance from cancer center, sicker quicker, outcomes, metastatic, treatment delay, non-small cell lung cancer

## Abstract

Introduction: The impact of diagnosis and treatment delay on outcomes in advanced non-small cell lung carcinoma (NSCLC) is not well understood. In this study, we examined the effect of the length of time to the first chemotherapy treatment initiation and the other factors affecting overall survival.

Methods: This retrospective study used data from the Institute of Clinical Evaluative Sciences and identified 4520 patients in Ontario who were diagnosed with stage IV NSCLC between 2007 and 2016, treated using chemotherapy. We adjusted the analysis for location (rural vs urban), gender, distance from the nearest cancer center, first chemotherapy treatment used, income, and age.

Results: Type of the chemotherapy, length of time to the first treatment, and distance from the nearest cancer center had a statistically significant impact on survival. Paclitaxel was associated with decreased risk of death compared to vinorelbine (Hazard Ratio (HR)=0.835, 95%CI 0.753-0.925), gemcitabine (HR=0.916, 95%CI 0.998-0.826), and docetaxel (HR=0.771, 95%CI 0.994-0.513). Every additional 10 km distance from the nearest cancer center was associated with a 0.5% increased risk of death (HR=1.005, 95%CI 1.000-1.010). A longer time to the first treatment was associated with increased survival. In fact, every 10 days increase in wait time was associated with a 0.5% decrease in the risk of death (HR=0.995, 95%CI 0.993-0.998).

Conclusion: Chemotherapy treatment using paclitaxel and living closer to the cancer center is associated with better survival. A longer time between diagnosis and treatment leading to better survival could perhaps be explained by patients on the "sicker" end of the spectrum receiving treatment sooner.

## Introduction

Lung cancer is the most diagnosed cancer worldwide and the leading cause of cancer death worldwide [1,2]. Indeed, lung cancer results in more deaths than breast, prostate, colorectal, and brain cancers combined [3]. Over 95% of all lung cancers are classified as either small cell lung cancer (SCLC) or non-small cell lung cancer (NSCLC) [4]. NSCLC accounts for 85% of all lung cancer cases and adenocarcinoma constitutes the most common type of NSCLC [4].

Staging of lung cancer is important for determining optimal medical management. Non-advanced NSLSC (stages I, II, III) can be managed curatively using either surgery, chemotherapy, radiation therapy, or a combination of therapies depending on the spread of the disease. Advanced NSLSC (stage IV) is associated with a worse prognosis; traditionally, patients with advanced NSCLC are treated with systemic therapy such as cytotoxic chemotherapy [5]. However, the use of chemotherapy in stage IV NSLSC is not curative, and only results in a small improvement in patient overall survival and quality of life [5]. Overall, patients diagnosed with advanced-stage NSLSC typically have a median survival of less than one year despite treatment and a five-year survival of less than 20% [6].

As such, it is important to investigate factors that affect a patient’s quality of care after diagnosis with late-stage NSLSC diagnosis so that we may ultimately make improvements in care that increase both the average quality of life and survival time after diagnosis in advanced lung cancer patients. Overall, these factors are not well understood.

One factor of interest in quality cancer care is the ‘timeliness of care’. It has been demonstrated that increased wait times decreased survival in lung cancer patients in Canada [7]. Other factors which may impact survival in stage IV NSLSC patients may include the proximity of the patient to the cancer centre, the type of chemotherapy agent they receive, their income, and whether they live in an urban or rural area. In this study, the primary aim was to investigate the relationship between these factors of care and the survival outcomes of patients diagnosed with stage IV NSLSC in Ontario, Canada. 

## Materials and methods

Data sources

This retrospective study used data from the Institute of Clinical Evaluative Sciences (ICES) and identified 4520 patients who were diagnosed with a stage IV Non-Small Cell Lung Carcinoma (NSCLC) between April 1, 2007, and Dec 31, 2016, in the province of Ontario in Canada. Patients who were treated with anything other than only first- and second-line NSCLC palliative chemotherapy (i.e., surgery and radiation) were excluded to ensure consistency when measuring outcomes. Large cell and squamous cell NSCLC patients were also excluded from the study cohort. Chemotherapy regimens included cisplatin/carboplatin plus one of gemcitabine, paclitaxel, docetaxel, pemetrexed, and vinorelbine. Some patients had switched regimens after the first line of treatment, and this was defined as second-line chemotherapy. Data for newer therapies like targeted therapy and immunotherapy were not available to be analyzed as administrative databases are not updated in a timely fashion to keep up with the advances in the treatments.

The date of diagnosis was defined as the date when stage IV NSCLC adenocarcinoma was confirmed histologically by a pathologist. Vital status (alive or dead) was accurate up until the follow-up cut-off date of December 31, 2017. Time from diagnosis to first treatment was defined as the number of days between the dates of diagnosis to the date of first-line chemotherapy administration. Time from the diagnosis to death or last follow-up was defined as the overall survival. 

Variables of interest

We examined the effect of the length of time from diagnosis to the first chemotherapy treatment on the survival of the patients from treatment initiation. Some variables of interest accounted for in our analysis included urban or rural location (rural defined as greater than 50 km from nearest cancer center), gender, distance from the nearest cancer center in kilometers, household income (in CAD$20,000 increments from CAD$0 to CAD$200,000 per annum), age, and first chemotherapy agent used.

Analysis

Data were analyzed using SAS version 9.4 (2013; SAS Institute Inc., Cary, North Carolina, United States). Upon ensuring the accuracy of data entry and statistical assumptions, descriptive statistics were performed to describe the sample characteristics. Kaplan-Meier and multivariate Cox regression analyses were performed, as appropriate, to address the outlined study objectives.

Cox regression analysis of the metastatic population was adjusted for general characteristics that were significantly different between groups. These included the patients' age, household income, and distance to the cancer center. All analyses were performed using a two-tailed α-value of 0.05, and either P≤0.05 or the 95%CI, was considered to indicate a statistically significant value.

## Results

The sample of 4520 patients was balanced with respect to income, with approximately 20% of patients in each of the five income brackets. Of this sample, 53% were male, 47% were female, 14% were from rural areas, and 86% were from urban areas. The most used chemotherapy agent was gemcitabine (63%), followed by paclitaxel (18.5%), vinorelbine (15.1%), docetaxel (2.6%), and pemetrexed (0.8%). The time to the first treatment had a median of 63 days (IQR: 40-98 days) while the distance from the nearest center had a median of 12 km (IQR: 5-48 km).

The Cox Proportional Hazards Regression Model analysis indicated that type of the chemotherapy, length of time to the first treatment, and distance from the nearest center had a statistically significant impact on survival, as shown in Table 1. Every additional 10 km increase in distance from the nearest cancer center was associated with a 0.5% increased risk of death (Hazard Ratio (HR) = 1.005, 95%CI 1.000-1.010). It was found that a longer diagnosis-treatment interval was better for survival. Every 10-day increase in the waiting time to initial treatment was associated with a 0.5% decrease in the risk of death (HR=0.995, 95%CI 0.993-0.998). 

**Figure 1:**
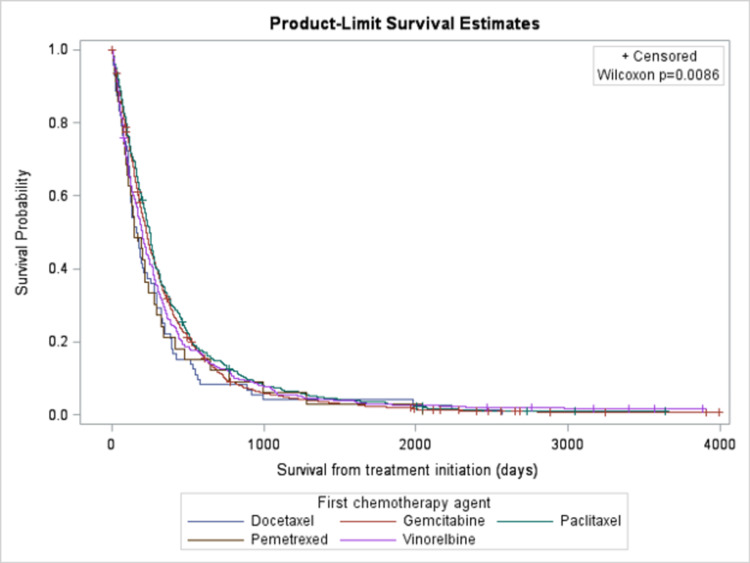
Kaplan-Meier Curve of Survival by Chemotherapy: patients treated with paclitaxel had a better survival compared to gemcitabine, vinorelbine, docetaxel, and pemetrexed

With regards to the type of chemotherapy used, paclitaxel had a lower HR and increased probability of survival compared to vinorelbine (HR = 0.835, 95%CI 0.753-0.925), docetaxel (HR = 0.771, 95%CI 0.513-0.994), and gemcitabine (HR = 0.916, 95%CI 0.826-0.998) (Table 1 and Figure 1). 

**Table 1:** Cox Proportional Hazards Regression Model: statistically significant factors impacting survival include chemotherapy used, distance to cancer center, and diagnosis-to-treatment time.

Compared factors	Hazard Ratio	95% Confidence Limits	
Differences among the chemo agents			
Docetaxel vs Gemcitabine	1.134	0.936	1.360
Docetaxel vs Paclitaxel	1.229	1.006	1.487
Docetaxel vs Pemetrexed	0.885	0.616	1.300
Docetaxel vs Vinorelbine	1.026	0.838	1.245
Gemcitabine vs Paclitaxel	1.084	1.002	1.174
Gemcitabine vs Pemetrexed	0.780	0.571	1.102
Gemcitabine vs Vinorelbine	0.904	0.832	0.985
Paclitaxel vs Pemetrexed	0.720	0.523	1.022
Paclitaxel vs Vinorelbine	0.835	0.753	0.925
Pemetrexed vs Vinorelbine	1.159	0.815	1.597
Distance to the nearest cancer center (per 10 km away)	1.005	1.000	1.010
Time to the first chemotherapy (per 10 days)	0.995	0.993	0.998

## Discussion

Principal findings

Our retrospective study aims to provide further clarification regarding the relationship between the diagnosis to treatment time for patients with stage IV NSCLC receiving chemotherapy treatment in Ontario from 2007 to 2016. Our principal finding shows a positive correlation between a longer diagnosis-to-treatment time and overall survival. For every 10 additional days between diagnosis and treatment, overall survival improved by 0.5% (95%CI 0.2%, 0.7%). This result adds to the existing literature, which has also described similar trends in other healthcare systems such as the United Kingdom and Australia. Termed "sicker quicker", previous authors suggest that patients who appear the most symptomatic receive treatment more urgently [8,9]. However, their survival is limited by the severity of their disease.

Hypothesis 1: Sicker Patients Receive Expedited Care But Still Die Quicker Due To Their Inherently Sicker State 

When considering why our study found that a longer diagnosis-treatment interval was better for survival, we hypothesized that the aforementioned “sicker quicker” principle might have been a contributing factor. However, it is difficult to declare conclusively as our data for each patient did not include patient comorbidities, sites of metastases, or the number of metastatic sites. This information would have allowed us to further categorize patients based on the severity and progression of their disease state and, in turn, examine whether it was correlated to their treatment time and subsequent survival time.

Other studies have suggested that those who experience shorter system delays may have poorer lung cancer survival because such patients may be at the "sicker" end of the spectrum present with symptoms that expedite their referral, diagnosis, and treatment, yet their survival is limited by the increased severity of their disease [8,10].

Hypothesis 2: "Sicker" Patients May Be Less Likely To Benefit From Chemotherapy

It is also possible that "sicker" patients are less likely to benefit from chemotherapy and are more likely to experience increased toxicity from chemotherapy which may, in fact, decrease their survival.

A key factor in evaluating the appropriateness of chemotherapy is the patient’s Eastern Cooperative Oncology Group performance status (PS). Studies from the 1980s established that poor PS (PS 3 and 4) is a strong predictor of poor survival, reduced response, and worsened toxicity to chemotherapy [11]. As such, the American Society of Clinical Oncology (ASCO) recommends against chemotherapy patients with a PS score of 3 or greater as the harms may outweigh the benefits [12]. However, many patients with poor PS continue to receive chemotherapy anyway and, in fact, chemotherapy in patients with poor PS seems to be increasing [8].

We postulate that symptomatic or "sicker" patients are more likely to start the treatment sooner but are less likely to derive benefit from palliative chemotherapy. Thus, it is possible that "sicker" appearing patients often experience shortened wait times for starting the treatment compared to healthier patients. 

However, a drawback to this hypothesis is recent evidence against the idea that poor PS patients are always harmed by cytotoxic chemotherapy. Patients with poor PS is a heterogeneous population, and those with poor PS attributable to high tumour burden alone may benefit from chemotherapy and other treatments rather than poor PS from multiple other comorbidities [13]. In contrast to studies from the past, recent studies have shown that modern less-toxic targeted therapies or immunotherapy may be associated with positive effects on survival for poor PS patients with stage IV NSLSC [11,14].

Clinical implications

Evidently, the decision-making process is complicated for both the patient and their medical team. The judicious use of chemotherapy only for patients who are likely to derive benefit has been shown to improve survival [6,15,16]. Additionally, enhanced communication of patient preferences may improve symptom management, helping reduce unintended harms of aggressive treatment leading to potential survival benefits [6]. Incorporating goals of care discussions, fostering better communication among the multidisciplinary care team, and setting realistic patient expectations for cancer treatment, may lead to improved patient selection for a treatment regimen most likely to derive benefit than harm [17].

Strengths and limitations of the study

The analysis of a large cohort of patients over a 10-year period from multiple cancer centers in Ontario is a strength of this study. To our knowledge, this study is the first to analyze the relationship between stage IV NSCLC diagnosis and treatment time and overall survival in Canada.

However, the use of ICES administrative data meant that we could not examine some potentially useful parameters including comorbidities and performance scores. Furthermore, our finding that a greater distance to the cancer center resulted in decreased overall survival would have been well complemented by comparing patient outcomes between urban academic cancer centers and more sparsely populated community cancer centers. Also, due to the lag time between data acquisition and analysis, we were not able to include patients who received targeted therapy and immunotherapy and compare their outcomes with patients who received chemotherapy.

Finally, the retrospective nature of our study could perhaps increase the likelihood of confounding, selection, and measurement bias and so the findings in the study should be interpreted with caution.

## Conclusions

In conclusion, our study revealed that there was a positive correlation between a longer diagnosis to treatment time and overall survival in stage IV NSCLC patients receiving chemotherapy in Ontario. Other factors we found to improve survival in our patient population included the type of chemotherapy regimen and living closer to the cancer center. These findings add to existing literature aiming to explain the “sicker quicker” phenomenon and prompt further analysis in order to find a definitive answer to this topic. Future studies can involve looking at diagnosis-to-treatment time stratified by performance score, as well as taking a deep dive into the clinical decision-making process when starting systemic therapy, especially as targeted therapies and immunotherapy have emerged as a promising treatment for advanced stage NSCLC.

## References

[1] Bray F, Ferlay J, Soerjomataram I, Siegel RL, Torre LA, Jemal A (2018). Global cancer statistics 2018: GLOBOCAN estimates of incidence and mortality worldwide for 36 cancers in 185 countries. CA Cancer J Clin.

[2] Usman Ali M, Miller J, Peirson L, Fitzpatrick-Lewis D, Kenny M, Sherifali D, Raina P (2016). Screening for lung cancer: a systematic review and meta-analysis. Prev Med.

[3] Siegel RL, Miller KD, Fuchs HE, Jemal A (2021). Cancer statistics, 2021. CA Cancer J Clin.

[4] (2021). American Cancer Society: Cancer information and resources. https://www.cancer.org/research/cancer-facts-statistics/all-cancer-facts-figures/cancer-facts-figures-2021.html..

[5] Parikh RB, Cronin AM, Kozono DE (2014). Definitive primary therapy in patients presenting with oligometastatic non-small cell lung cancer. Int J Radiat Oncol Biol Phys.

[6] Lammers A, Slatore CG, Fromme EK, Vranas KC, Sullivan DR (2019). Association of early palliative care with chemotherapy intensity in patients with advanced stage lung cancer: a national cohort study. J Thorac Oncol.

[7] Kasymjanova G, Small D, Cohen V (2017). Lung cancer care trajectory at a Canadian centre: an evaluation of how wait times affect clinical outcomes. Curr Oncol.

[8] Forrest LF, Adams J, White M, Rubin G (2014). Factors associated with timeliness of post-primary care referral, diagnosis and treatment for lung cancer: population-based, data-linkage study. Br J Cancer.

[9] Malalasekera A, Blinman PL, Dhillon HM (2018). Times to diagnosis and treatment of lung cancer in New South Wales, Australia: A Multicenter, Medicare Data Linkage Study. J Oncol Pract.

[10] Myrdal G, Lambe M, Hillerdal G, Lamberg K, Agustsson Th, Ståhle E (2004). Effect of delays on prognosis in patients with non-small cell lung cancer. Thorax.

[11] Salloum RG, Smith TJ, Jensen GA, Lafata JE (2012). Survival among non-small cell lung cancer patients with poor performance status after first line chemotherapy. Lung Cancer.

[12] Burris HA 3rd (2020). Correcting the ASCO position on phase I clinical trials in cancer. Nat Rev Clin Oncol.

[13] Kancharla H, Gundu N, Pathak N (2020). Cytotoxic chemotherapy in advanced non-small cell lung cancer with poor performance status: a retrospective analysis from routine clinical practice. Curr Probl Cancer.

[14] Leong SS, Toh CK, Lim WT (2007). A randomized phase II trial of single-agent gemcitabine, vinorelbine, or docetaxel in patients with advanced non-small cell lung cancer who have poor performance status and/or are elderly. J Thorac Oncol.

[15] Grunfeld E, Watters JM, Urquhart R (2009). A prospective study of peri-diagnostic and surgical wait times for patients with presumptive colorectal, lung, or prostate cancer. Br J Cancer.

[16] Schiller JH, Harrington D, Belani CP (2002). Comparison of four chemotherapy regimens for advanced non-small-cell lung cancer. N Engl J Med.

[17] Jang RW, Le Maître A, Ding K (2009). Quality-adjusted time without symptoms or toxicity analysis of adjuvant chemotherapy in non-small-cell lung cancer: an analysis of the National Cancer Institute of Canada Clinical Trials Group JBR.10 trial. J Clin Oncol.

